# Supplementary material for: Availability and factors influencing community level handwashing facility in Ethiopia: Implication for prevention of infectious diseases
Source: PLoS One. 2021 Jan 19;16(1):e0243228. doi: 10.1371/journal.pone.0243228 (PMC7815131; doi:10.1371/journal.pone.0243228)
Supplement: Attachment — Submitted filename: Review comments-PLOSONE-Global Health_2020.pdf [file pone.0243228.s001.pdf]

Review Comments: Availability and factors influencing community level handwashing facility in Ethiopia: An implication for prevention of infectious diseases

10/08/2020

Thank you for the opportunity to review this manuscript. It was very well-written manuscript with a logical and easy to follow structure and I enjoyed reading it. I believe it is an important contribution to the WASH literature. I have only a few concerns:

1. Title: I would recommend deletion of the word '*an*' to read: ***Availability and factors influencing community level handwashing facility in Ethiopia: implication for prevention of infectious diseases***
2. Introduction: I would recommend the use more recent literature in the introduction. I believe there more recent literature that can replace references 1, 4,5 and 6 [e.g. review by Willmott, M et al 2015]
3. Methods: Line 115 I would recommend deletion of the word "recent" and instead say… formed in 2007
4. Results:
    i. The concepts of ***hot and cold spots*** used in line 173 page 10 need to be defined
    ii. Table 3 appearing on page 10-12 may need to be reduced or formatted to fit in one page in its current format, it difficult to follow
    iii. On page 10, line 182, the 54.81% of household heads with no formal education; it is not clear what the denominator is. Are we referring to the female headed household or the total sample
5. Discussion: The discussion section is well written, however:
    i. This section would flow better if the authors started by summarizing the key results before starting the implication of the results
    ii. I would recommend that the authors include a discussion on relationship between availability of hand washing facility and household wealth which would allow more balance conclusions and recommendation. As it stands now the authors seem to suggest that improved awareness equals action/behavior (installation of hand washing facility), although they do acknowledge acknowledge that education as necessary but insufficient for changing behavior [line240].
