# Supplementary material for: Availability and factors influencing community level handwashing facility in Ethiopia: Implication for prevention of infectious diseases
Source: PLoS One. 2021 Jan 19;16(1):e0243228. doi: 10.1371/journal.pone.0243228 (PMC7815131; doi:10.1371/journal.pone.0243228)
Supplement: Attachment — Submitted filename: Response to Reviewers.docx [file pone.0243228.s002.docx]

**Response to Reviewers**

**We are grateful to reviewers for the time and professional contribution!**

Review Comments: Availability and factors influencing community level handwashing facility in Ethiopia: An implication for prevention of infectious diseases

10/08/2020

Thank you for the opportunity to review this manuscript. It was very well-written manuscript with a logical and easy to follow structure and I enjoyed reading it. I believe it is an important contribution to the WASH literature. I have only a few concerns:

Response: Thank you very much!

1. Title: I would recommend deletion of the word ‘an’ to read: Availability and factors influencing community level handwashing facility in Ethiopia: implication for prevention of infectious diseases

Response: comment accepted, and the title is changed to: Availability and factors influencing community level handwashing facility in Ethiopia: implication for prevention of infectious diseases

2. Introduction: I would recommend the use more recent literature in the introduction. I believe there more recent literature that can replace references 1, 4,5 and 6 [e.g. review by Willmott, M et al 2015]

Response: comments accepted and most of the references updated to the latest available/suitable evidence

3. Methods: Line 115 I would recommend deletion of the word “recent” and instead say… formed in 2007

Response: comment accepted and changed for the 2007 population census

4. Results:
i. The concepts of hot and cold spots used in line 173 page 10 need to be defined

Response: important comment! It is corrected/defined as:

Hot spot areas of handwashing facilities (clusters where relatively large number of households that had basic handwashing facilities recorded) were detected in the capital city, Addis Ababa, and in a few places of part of Oromia region surrounding Addis Ababa (99% confidence hot spot areas).

Cold spot areas of handwashing facilities (clusters where relatively small number of households that had basic handwashing facilities recorded) were observed in Amhara, Gambela and SNNP regions (99% confidence cold spot areas).

ii. Table 3 appearing on page 10-12 may need to be reduced or formatted to fit in one page in its current format, it difficult to follow

Response: We agree with the reviewer’s comments, but we thought it will be reduced to one page during production and the current line spacing was 2 pt. Now we changed the spacing to 1.15 pt and this fit to a page. We hope will not be more than a page in the final (published) paper.

iii. On page 10, line 182, the 54.81% of household heads with no formal education; it is not clear what the denominator is. Are we referring to the female headed household or the total sample

Response: comment accepted and corrected as: One-fourth of households were headed by females, and of the total sample (households headed by male and female), 54.81% of household heads had no formal education.

5. Discussion: The discussion section is well written, however:
i. This section would flow better if the authors started by summarizing the key results before starting the implication of the results

Response: comment accepted and the first paragraph of the discussion section modified as follow:

This study set out with the aim of assessing the availability and factors influencing community level handwashing facilities in Ethiopia. Overall, our finding revealed that more than 90% of households had no basic handwashing facilities. This study also highlighted factors that were associated with the availability of basic handwashing facilities at the community-level. To improve the health status of its citizen in an equitable manner, Ethiopia has implemented successive Health Sector Development Plans (HSDPs) with the key concept of ensuring community ownership and enabling them to produce their own health. One of the globally noted components of Ethiopian HSDPs is health extension program (HEP), which has a total of 16 packages, of which 7 are environmental health related. Despite such tremendous efforts made by the government, the current study found that fewer than 10% of households in Ethiopia had basic handwashing facilities. This implied that Ethiopia is far behind the Sustainable Development Goals (SDGs) ambition to achieving access to sanitation and hygiene services by the year 2030, and continued to suffer from infectious diseases which could potentially be prevented by washing hands with water and soap.

ii. I would recommend that the authors include a discussion on relationship between availability of hand washing facility and household wealth which would allow more balance conclusions and recommendation. As it stands now the authors seem to suggest that improved awareness equals action/behavior (installation of hand washing facility), although they do acknowledge acknowledge that education as necessary but insufficient for changing behavior [line240].

Response: We agree that household wealth has direct and positive correlation with the availability of handwashing facility, and we mentioned that in our discussion. Now, based on the reviewers’ comment we feel that we need to modify the existing discussion section on this sub-topic, so that, we made slight modification on our previous discussion as follow:

Ethiopian DHS collects water source, sanitation facilities, type of flooring, electricity, the number of rooms per person living in the house, and possession of radio, television, phone (landline or cell phone), motorcycle and car. These household assets used to compute household wealth index using a principal component analysis. Based on this, compared to households in the first wealth quintile, those in the second, third, fourth and fifth quintiles were more likely to have basic handwashing facilities. This result may be explained by the fact that this household owned assets-based wealth index helps to show the status of households in terms of income and livelihood standards, which could in turn determined the household’s ability to install basic handwashing facilities. The current result is in line with existing research-based evidence where a positive correlation between economic status and handwashing facilities at the household and the country level were reported. This finding has important implications for developing targeted sanitation- and hygiene-related subsidies in the community.

**In summary**

- We thought we have addressed all the concerns raised by our editor and reviewers. The issues raised by the first reviewer are all incorporated in the document.
- As it can be seen the submitted files, all editorial and formatting issues addressed.
